# *doublesex* Controls Both Hindwing and Abdominal Mimicry Traits in the Female-Limited Batesian Mimicry of *Papilio memnon*

**DOI:** 10.3389/finsc.2022.929518

**Published:** 2022-07-12

**Authors:** Shinya Komata, Chung-Ping Lin, Haruhiko Fujiwara

**Affiliations:** ^1^Department of Integrated Biosciences, Graduate School of Frontier Sciences, The University of Tokyo, Kashiwa, Japan; ^2^Department of Life Science, National Taiwan Normal University, Taipei, Taiwan

**Keywords:** Batesian mimicry, *Papilio memnon*, electroporation-mediated gene knockdown, abdominal mimicry, female-limited polymorphism, *doublesex* (*dsx*), swallowtail butterfly, supergene

## Abstract

*Papilio* butterflies are known to possess female-limited Batesian mimicry polymorphisms. In *Papilio memnon*, females have mimetic and non-mimetic forms, whereas males are monomorphic and non-mimetic. Mimetic females are characterized by color patterns and tails in the hindwing and yellow abdomens. Recently, an analysis of whole-genome sequences has shown that an approximately 160 kb region of chromosome 25 is responsible for mimicry and has high diversity between mimetic (*A*) and non-mimetic (*a*) alleles (highly diversified region: HDR). The HDR includes three genes, *UXT*, *doublesex* (*dsx*), and *Nach-like*, but the functions of these genes are unknown. Here, we investigated the function of *dsx*, a gene involved in sexual differentiation, which is expected to be functionally important for hindwing and abdominal mimetic traits in *P*. *memnon*. Expression analysis by reverse transcription quantitative PCR (RT-qPCR) and RNA sequencing showed that mimetic *dsx* (*dsx-A*) was highly expressed in the hindwings in the early pupal stage. In the abdomen, both *dsx-A* and *dsx-a* were highly expressed during the early pupal stage. When *dsx* was knocked down using small interfering RNAs (siRNAs) designed in the common region of *dsx-A* and *dsx-a*, a male-like pattern appeared on the hindwings of mimetic and non-mimetic females. Similarly, when *dsx* was knocked down in the abdomen, the yellow scales characteristic of mimetic females changed to black. Furthermore, when *dsx-a* was specifically knocked down, the color pattern of the hindwings changed, as in the case of *dsx* knockdown in non-mimetic females but not mimetic females. These results suggest that *dsx-a* is involved in color pattern formation on the hindwings of non-mimetic females, whereas *dsx-A* is involved in hindwing and abdominal mimetic traits. *dsx* was involved in abdominal and hindwing mimetic traits, but *dsx* expression patterns in the hindwing and abdomen were different, suggesting that different regulatory mechanisms may exist. Our study is the first to show that the same gene (*dsx*) regulates both the hindwing and abdominal mimetic traits. This is the first functional analysis of abdominal mimicry in butterflies.

## Introduction

**Figure 1:**
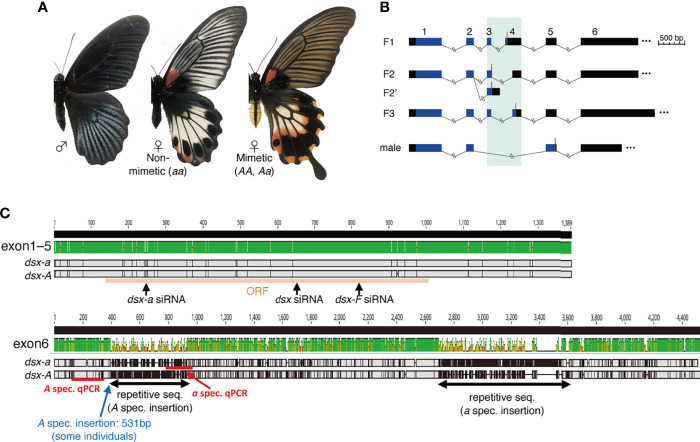
**(A)** Wing and abdominal patterns of *Papilio memnon* males and non-mimetic and mimetic females. **(B)** Schematic of *doublesex* (*dsx*) isoform in *P*. *memnon*. Female isoforms are divided into three types by start to stop codon sequences (F1, F2, F3), and F2 is divided into two types by 3’-untranslated region (UTR) sequences (F2, F2’). There is one male isoform type. The region shown in black is the UTR, and the region shown in blue is the open reading frame (ORF). Red bars indicate the position of the stop codon. The numbers above indicate the exon numbers (exon 1 to exon 6). Exons 3 and 4, which are highly variable among isoforms, are highlighted with a green background. **(C)** Alignment visualization of the *dsx* F1 isoform and the target positions of the small interfering RNA (siRNA) and quantitative PCR (qPCR) primers used in this study. There are few SNPs from exon 1 to exon 5, and *dsx-A* and *dsx-a* are diversified in exon 6. In exon 6, only some individuals had a 531 bp insertion. The top image shows the sequence similarity between *dsx-A* and *dsx-a* visualized by Geneious Prime (2022.0). The middle shows F1 from *dsx-a*, and the bottom shows F1 from *dsx-A*. Areas in gray indicate the sequence identity between *dsx-a* and *dsx-A*, black indicates single nucleotide polymorphisms (SNPs), and horizontal bars indicate deletions.

Batesian mimicry is a phenomenon in which a species that is non-toxic and tasty to predators, such as birds (mimic), escape predation by mimicking the appearance, shape, and behavior of a toxic and tasteless species (model) (1). The most interesting and oldest known example of Batesian mimicry is the female-limited Batesian mimicry in swallowtail butterflies (2, 3). In *Papilio memnon*, only females possess mimetic and non-mimetic forms, and males are monomorphic and non-mimetic. In *P*. *memnon*, mimetic females are characterized by morphological traits, such as color patterns and tails in the hindwing and yellow abdomens (**Figure 1A**), and they mimic the butterflies *Pachliopta aristolochiae*, *Atrophaneura coon*, and *Atrophaneura polyeuctes* (4–6). Mimetic females mimic the model set of these three traits, and according to Mendelian inheritance (mimetic type *A* is dominant and non-mimetic type *a* is recessive), no recombinant forms occur, and the mimetic and non-mimetic forms are maintained, leading to the supergene hypothesis (4, 5). In other words, it is hypothesized that there are genes that control each trait and that these genes are tightly linked together to control complex mimicry *via* multiple traits.

Recently, a whole-genome analysis was performed in *P*. *memnon*. It showed that an approximately 160 kb region on chromosome 25 is a highly diversified region (HDR) between mimetic and non-mimetic forms and is responsible for mimicry (7–9). The HDR contained the 5’-untranslated region (UTR) of *ubiquitously expressed transcript (UXT*), the full length of *doublesex* (*dsx*), and most of *Nach-like* (8). In the closely related species *P*. *polytes*, Batesian mimicry is also limited to females (mimetic (*H*) and non-mimetic (*h*) types in females), and almost the same region of chromosome 25 containing the *UXT*, *U3X* and *dsx* genes was found to be responsible for mimicry (10, 11). A region of approximately 130–160 kb, almost the same region in both *P*. *memnon* and *P*. *polytes*, is the cause of mimicry (HDR), and recombination is suppressed in this region. However, there is an inversion in this region at the *H* and *h* alleles in *P*. *polytes* not in *P*. *memnon* (8–12).

The gene within the HDR that appears to be crucial is *dsx*. DSX is a transcription factor involved in sexual differentiation, and its domain structure is conserved between vertebrates and insects (13, 14). *dsx* was originally known to be involved in the control of important traits related to sexual differentiation and reproduction during early development. However, recent reports have shown that it is involved in regulating sex-specific traits, such as horns and mandibles in beetles and abdominal coloration and combs in *Drosophila* (14–17). *dsx* has also been shown to be important for mimetic color pattern formation in *P*. *polytes* through functional analysis using *in vivo* electroporation-mediated RNA interference (RNAi) (11, 18, 19). The knockdown of mimetic *dsx* (*dsx-H*) in the hindwings of mimetic females results in a reduction in the red spots characteristic of the mimetic form and a significant change in the pale-yellow spots from the mimetic pattern to the non-mimetic pattern (11, 18, 19). However, Komata et al. (20) showed that not only *dsx* but also *UXT*, *sir2*, and a noncoding RNA *U3X* are involved in hindwing color pattern formation in *P*. *polytes*. While *dsx* causes large phenotypic changes in mimetic color pattern formation limited to females, *UXT* and *sir2* cause relatively small phenotypic changes in mimetic color pattern formation and may act as modifying factors to make the mimetic phenotype more similar to the model. *U3X* is a noncoding RNA that may regulate the expression of other genes (20). In *P*. *memnon*, the function of genes within the HDR has not been investigated by functional analysis. In this study, we investigated the function of *dsx* in the mimetic traits of *P*. *memnon*. We first identified *dsx* isoforms, analyzed *dsx-A* and *dsx-a* expression, and evaluated their functions by RNAi using *in vivo* electroporation.

One of the notable features of *P*. *memnon* mimetic females is the yellow abdomen. In the subgenus *Menelaides*, which includes *P*. *memnon* and *P*. *polytes*, *P*. *aegeus* and *P*. *rumanzovia* also possess female-limited mimicry (9, 21). In *P*. *memnon*, mimetic females have yellow abdomens, whereas males and non-mimetic females are black with no pattern (**Figure 1A**). In contrast, *P*. *polytes* and *P*. *rumanzovia* show a polymorphism in the wing color pattern of females, but males and non-mimetic and mimetic females have black abdomens and are not polymorphic (22–24). For *P*. *aegeus*, as in *P*. *memnon*, the abdomen is yellow in only one female form (23, 24). Although a few swallowtail butterflies display a sexual dimorphism or intraspecific polymorphism in abdominal coloration, *P*. *jordani*, considered a member of the *Menelaides* subgenus, has a whitish abdomen only in females (22, 24). *P*. *jordani* is a rare species found only in Sulawesi, and its molecular mechanisms and phylogenetic relationships are unclear. In the genus *Papilio*, the tiger swallowtail butterflies, *P*. *glaucus*, show both mimetic and non-mimetic types in females and female-limited mimetic polymorphisms, with mimetic types mimicking *Battus philenor* by darkening the wings and abdomen entirely (24). Males and non-mimetic females have yellow and black color patterns on the wings and abdomen. Although the gene controlling the mimicry polymorphism, which is limited to females, is not known for *P*. *glaucus*, there are differences in the molecular mechanisms involved in the melanin synthesis system between mimetic and non-mimetic females and males, with yellow papillochrome being synthesized in the wild type. However, in mimetic females, the yellow papillochrome is replaced by a black melanin pigment (25–28). Differences in the synthesized pigments are associated with differences in gene expression, such as *dopa decarboxylase* (*DDC*) and *N-β-alanyl-dopamine-synthase* (*BAS*) (25–27).

The molecular mechanisms of wing color patterns have been investigated in many butterflies (12, 29–36), but abdominal color pattern polymorphisms have not been examined to date. Therefore, we investigated the involvement and function of *dsx* in hindwing color pattern and abdominal yellow in *P*. *memnon*. So far, we have investigated gene function in *P*. *polytes* by introducing small interfering RNAs (siRNAs) at arbitrary sites using *in vivo* electroporation and partial knockdown (37, 38). In particular, siRNA was introduced into the hindwing immediately after pupation to perform knockdown, which can also be used to introduce siRNA into the abdomen. siRNA can be introduced into the abdomen of fifth instar larvae during the wandering stage by electroporation as in the hindwing to perform knockdown. In *P*. *polytes*, *TH* and *laccase 2* knockdown has been reported in the abdomen, and these genes are involved in the synthesis of the black pigment in the adult abdomen (39).

In this study, we identified and described *dsx* isoforms in *P*. *memnon*. *dsx* has been reported to produce female and male isoforms by alternative splicing in insects, with multiple additional isoforms in females. In *P*. *memnon* and *P*. *polytes*, the *dsx* gene consists of six exons, and in *P*. *polytes*, a close relative of *P*. *memnon*, there are three major isoforms in females and one in males (8, 11). Only female isoform 3 (F3) is involved in female mimetic color pattern formation (20). Since isoforms have not been clearly identified in *P*. *memnon* before, we described multiple isoforms using RNA sequencing (RNA-seq) and PCR. In particular, since *dsx-A* and *dsx-a* have few base substitutions in the open reading frame (ORF) region, it was necessary to clarify the structure and sequence of the isoforms to design *dsx-A*- and *dsx-a*-specific quantitative PCR (qPCR) primers in the UTR region, which varies between *dsx-A* and *dsx-a*. It has also been suggested that there are allele-specific insertions and repeat sequences in exon 6, which contain only the UTR, although there are large discrepancies between *dsx-A* and *dsx-a* (8).

Next, we examined *dsx* expression using reverse transcription qPCR (RT-qPCR) and RNA-seq. In *P*. *polytes*, *dsx-H* is highly expressed, especially on the second day after pupation (P2), and is thought to be involved in mimetic color pattern formation (11, 40). In addition, *dsx-H* and *dsx-h* show contrasting expression patterns. *dsx-H* is highly expressed in the early pupal stage and *dsx-h* in the late pupal stage (20). This may be due to *dsx-H*-specific expression regulation by an enhancer within the HDR and/or a long noncoding RNA (*U3X)*. Therefore, we also examined the expression of *dsx-A* and *dsx-a* in *P*. *memnon* by RT-qPCR in the entire hindwing and dorsal surface of the fifth abdominal segment (A5, the area where the abdomen turns yellow in adult mimetic females) at the second day after pupation (P2), the 5th day after pupation (P5), and the 10th day after pupation (P10). For comparison, we also examined *dsx-H* and *dsx-h* expression in the A5 of *P*. *polytes*, mimetic females that do not have a mimetic trait in the abdomen. We also performed RNA-seq in the hindwings of mimetic and non-mimetic females at P2 and the 7th day after pupation (P7) and compared the expression levels of *dsx-A*, *dsx-a*, and the three isoforms in females.

Finally, we investigated the function of *dsx* by electroporation-mediated RNAi. siRNA was injected into the hindwing immediately after pupation or into the A5 during the wandering stage of the late fifth larval instar. RNAi was performed by introducing three siRNAs separately: siRNA targeting sequences common to *dsx-A*, *dsx-a*, and all isoforms (*dsx-common* siRNA), siRNA targeting sequences found only in *dsx* female isoforms (*dsx-F* siRNA), and siRNA targeting only *dsx-a* (*dsx-a* siRNA). Since *dsx* was originally involved in sexual differentiation, we expected *dsx* knockdown introduced by *dsx-common* siRNA to result in a male-like phenotype in mimetic and non-mimetic females. In addition, *dsx-F* siRNA would show phenotypic changes similar to those of *dsx-common* siRNA. Although we were unable to design siRNAs targeting only *dsx-A* because we could not find a suitable sequence, we thought that knockdown using *dsx-a* siRNA would allow us to consider the functions of *dsx-A* and *dsx-a*.

## Materials and Methods

### Butterfly Collection and Rearing

We captured adult *P*. *memnon* females from October 16–20, 2018, and May 11–15, 2019, in Hualien, Eastern Taiwan (23°59N, 121°32E). The females laid eggs in the laboratory, and the hatched larvae were reared at 25°C under an LD 16:8 photoperiod and used for the experiments. We fed the larvae *Citrus* leaves (Rutaceae) and the adults a sports drink (Calpis, Asahi, Japan).

### Sequence of *dsx* Isoforms and *A* and *a* Alleles by RNA-seq and PCR

Nishikawa et al. (11) identified three isoforms derived from mimetic and non-mimetic alleles in females and one in males, respectively, in *P. polytes*. However, they were not clearly described in *P*. *memnon*. Therefore, we searched for each isoform using existing RNA-seq data (7, 8) and performed PCR and electrophoresis to confirm the results. First, RNA-seq read data from the entire hindwings of four mimetic (*dsx* genotype: *Aa*) females, one *aa* male, and three *Aa* males seven days after pupation (P7) were used to explore *dsx* sequences by *de novo* assembly and mapping to a reference genome. **Table S1** shows the P7 RNA-seq data. We performed *de novo* assembly using Trinity (v2.8.3) (41) with default settings and BLAST 2.9.0+ (42) to search *dsx* sequences. The F1 isoform (ORF only sequence) (8) of *dsx* in *P*. *memnon* was used as the query sequence. In addition, we used the STAR (version 2.7.1a) (43) and Cufflinks (v2.2.1) (44) pipelines with default settings to detect transcripts by mapping to the *P*. *memnon* reference genome (BioProject: PRJDB5519). Existing gene annotation information was not used as a guide in this process. We also mapped and searched for transcripts by default in HISAT2 (version 2.1.0) (45) and StringTie (v1.3.6) (46) pipelines. In addition to STAR/Cufflinks, we used the *P*. *memnon* reference genome for mapping, and existing gene annotations as a guide (BioProject: PRJDB5519) for HISAT2/StringTie analysis. Thus, we confirmed that there are three major isoforms in females and one isoform in males, but the isoforms detected were biased by differences in RNA-seq read data analysis methods and the sample (e.g., no individual had all three female isoforms detected at the same time) (**Table S2**). Therefore, we used PCR to confirm the presence of all isoforms. We extracted mRNA from the hindwings at P2, synthesized cDNA, conducted PCR using KOD FX Neo (Toyobo, Osaka, Japan), and performed electrophoresis. mRNA extraction and cDNA synthesis were performed as described for RT-qPCR.

### RT-qPCR

We performed RT-qPCR to examine the expression patterns in the hindwing and abdomen of *dsx-A* and *dsx-a* individuals. The entire hindwing and dorsal surface of the A5 segment were sampled for RNA extraction on pupal days 2 (P2), 5 (P5), and 10 (P10), and RNA extraction was performed using the TRI reagent (Sigma) in the same manner as mentioned in Nishikawa et al. (11) and Iijima et al. (18). DNase I (TaKaRa, Japan)-treated RNA was subjected to cDNA synthesis using a Verso cDNA Synthesis Kit (Thermo Fisher Scientific). qPCR was performed using Power SYBR^®^ Green Master Mix (Thermo Fisher Scientific) and QuantStudio 3 (Applied Biosystems) (18). The genotype of *dsx* in the sample was confirmed by qPCR using cDNA synthesized from mRNA of the pupal hindwings. The genotypes of individuals can be distinguished by confirming amplification using primers specific for *dsx-A* or *dsx-a* (7, 8). The *dsx-A* and *dsx-a* specific primers are the same primer used for examining their expression patterns (**Table S3**). In the pupal stage, the shape of the hindwing (with or without tail) can be also distinguished between mimetic and non-mimetic females. We used *P*. *memnon* hindwing and abdominal samples from 30 individuals. Similarly, we collected 26 P. *polytes* RNA samples from the dorsal side of A5 for comparison. The number of samples of *P*. *polytes* mimetic females at P10 is two, then we removed the P10 of mimetic female from statistical analysis in *P*. *polytes*. *Ribosomal protein L3* (*RpL3*) was used as an internal standard, and **Table S3** lists the primers used.

We explored the effects of stage and genotype/sex using a generalized linear model (GLM) with a normal distribution. Tukey’s *post hoc* tests were used to detect differences between groups using the “glht” function in the R package multcomp (47, 48).

### RNA-seq

We performed RNA-seq expression analysis to compare *dsx-A*, *dsx-a*, and *dsx* isoform expression levels. In addition to the RNA-seq read data in the P7 hindwings obtained in previous studies (7, 8), we adjusted the mRNA from the entire hindwing of five new P2 mimetic females, four P2 non-mimetic females, and four P7 and P8 non-mimetic females. The four non-mimetic females included one P8 and three P7 individuals, but P7 and P8 expression levels did not differ significantly. Hence, they were treated as samples in the mid-pupal stage and were referred to as P7 individuals in the results. The developmental period of *P*. *memnon* pupae is approximately 13 to 14 days at 25°C (49). Thus, P7 and P8 are in the mid-pupal stage.

**Table S1** lists the samples used in the RNA-seq experiments. The extracted and DNase I (TaKaRa, Japan)-treated RNA was sent to Macrogen Japan Corporation for library preparation using TruSeq stranded mRNA (paired-end, 101 bp) and sequenced using the Illumina platform. The obtained RNA-seq reads were quality-checked using FastQC (version 0.11.9) (50), mapped using Bowtie 2 (version 2.4.4) (51), and the number of reads was counted using SAMtools (version 1.14) (52). Based on the number of reads, the fragments per kilobase of transcript per million mapped reads (FPKM) value was calculated (as the number of mapped reads/gene length(bp)/total number of reads × 10^9^).

We mapped the *dsx-A*- and *dsx-a*-derived sequences of the three female *dsx* isoforms (F1, F2, and F3). Exon 6 of *dsx* had a repetitive sequence found on other chromosomes in the genome. Hence, we mapped the sequences of exons 1–5 and the first part of exon 6 (about 400 bp). The *UXT* contained within the HDR was also mapped using the full-length *UXT*. Based on data from Iijima et al. (8), we obtained sequence data for *UXT* from the *P*. *memnon* reference genome (BioProject: PRJDB5519). We analyzed differences in expression between alleles and P2 and P7 using a GLM, Tukey’s *post hoc* tests in the same way to RT-qPCR.

### *In Vivo* Electroporation-Mediated RNAi

Three siRNAs targeting *dsx* were used in this study (**Figure 1C**, **Table S3**). One was a siRNA common to *dsx-A*, *dsx-a*, and all isoforms, and the same siRNA was used to knockdown *dsx* in the closely related species *P. polytes* sequence (11). The other two were designed using siDirect (http://sidirect2.rnai.jp/) with *dsx-a* siRNA and *dsx-F* siRNA for the specific knockdown of *dsx-a* and the female isoform, respectively.

The target sequences were blasted against the predicted gene and genome (BioProject: PRJDB5519) sequences in *P*. *memnon* to confirm that the sequences were highly specific, especially for the target genes. The designed siRNA was synthesized by FASMAC Co. Ltd. (Kanagawa, Japan). The RNA powder received was dissolved in nuclease-free water (Thermo Fisher, Ambion), adjusted to 500 μM, and stored at -20°C. **Table S3** lists the siRNA sequence used. A glass capillary (Narishige, GD-1 Model, glass capillary with filament) was processed into a needle shape by heating at HEATER LEVEL 68 using a puller (Narishige, PP-830 Model). Capillaries were then filled with siRNA. The siRNA concentration was then adjusted to 250 μM. The capillary was filled with siRNA, and 4 μl of siRNA was injected into the left hindwing or abdomen (A5) under a stereomicroscope using a microinjector (FemtoJet, Eppendorf). Then, siRNA was introduced into only the positive pole side of the electrode by applying a voltage (5 square pulses of 7.5 V, 280 ms width) using an electroporator (Cellproduce, Electrical Pulse Generator CureGine). A phosphate-buffered saline (PBS) gel (20×PBS: NaCl 80 g, Na_2_HPO_4_ 11 g, KCl 2 g, KH_2_PO_4_ 2 g, DDW 500 ml; 1% agarose) was placed on the dorsal side of the hindwing, and a drop of PBS was deposited on the ventral side of the hindwing. A drop of PBS gel (0.3% agarose) was put on both sides of the A5 segment for abdominal RNAi. The detailed method follows that described in previous studies (37, 38). The Supplementary Figures collectively report all the individuals who performed the functional analyses.

## Results

### *dsx* Isoforms in *P*. *memnon*

We examined the *dsx* isoforms of *P*. *memnon* using RNA-seq read data from previous studies (7, 8) and found that, like *P*. *polytes*, there were three types of female isoforms based on the ORF type and one male isoform (**Figure 1B**). Female isoform F2 had two isoforms, depending on the sequence after the terminal codon (i.e., UTR) (**Figure 1B**). *P*. *polytes* also varied in F2 (11). As for exon 6, the obtained sequences differed in length depending on the contig, ranging from approximately 1000 bp to nearly 8000 bp (**Table S2**). Exon 6 has been found to contain many repetitive sequences (8), and it is unknown if the contig obtained by RNA-seq assembly is present as an actual transcript. Repetitive sequences may also yield contigs that do not exist as transcripts. We designed primers in a region approximately 1200 bp from the 5’ side of exon 6 and performed PCR. We found that exon 6 was approximately 1200 bp long in all female isoforms (**Figure S1**). Interestingly, some mimetic females (*Aa*) had a band longer than the expected sequence length, and Sanger sequencing revealed a 531 bp insertion of approximately 350 bp from the 5’ side of exon 6 (**Figure 1C**, **Figure S1**). It has been shown that the mimicry HDR contains more repetitive sequences, such as transposons, than other genomic regions (8, 12), and it is likely that repetitive sequences are similarly accumulated for exon 6 in *dsx*. Based on the above, we could describe the isoform of *dsx* in *P*. *memnon*, but there is a large diversity of exon 6 sequences between *dsx-A* and *dsx-a*. Furthermore, there may be variations among individuals, and such variation may be involved in polymorphism maintenance. In *P*. *memnon*, recombination is suppressed despite the absence of chromosomal inversion, and it has been suggested that the insertion of transposons or other repetitive sequences may contribute to recombination suppression (8, 12). Insertions were observed only in some individuals, indicating that transposons and other insertions are more likely to occur and accumulate in the HDR.

### Expression Levels of *dsx-A*, *dsx-a*, and the Three Isoforms

**Figure 2:**
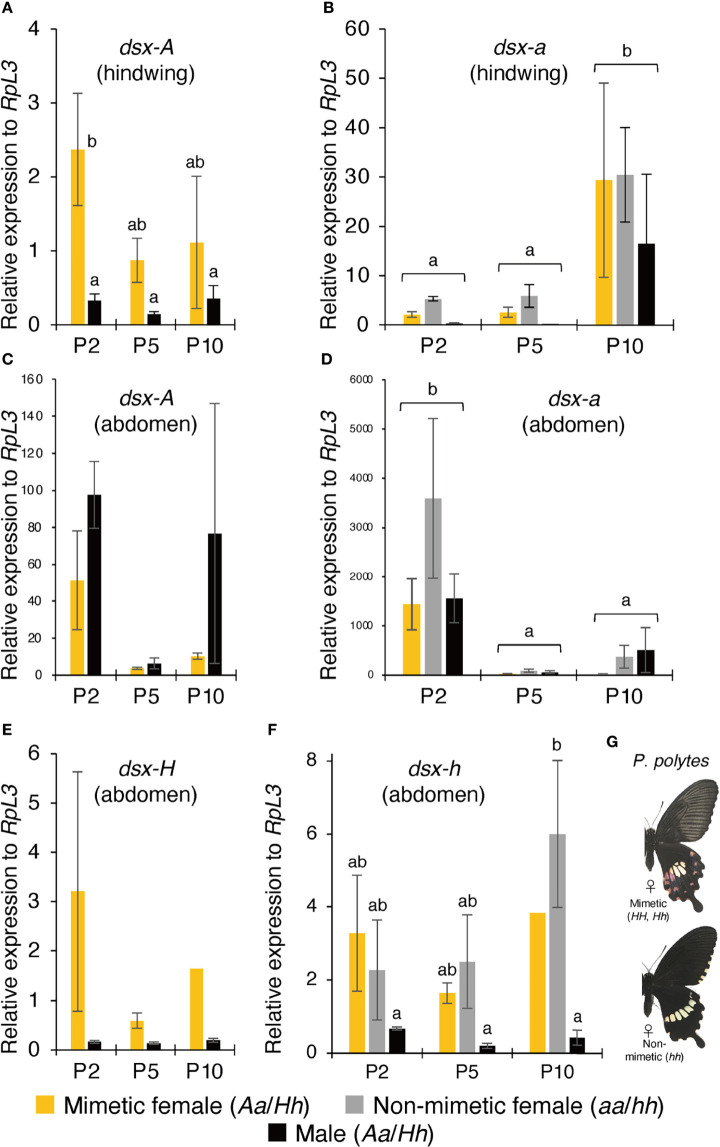
**(A–D)** Gene expression levels in mimetic (*dsx* genotype: *Aa*) and non-mimetic (*dsx* genotype: *aa*) *Papilio memnon* females and males (*dsx* genotype: *Aa*) at two (P2), five (P5), and ten (P10) days after pupation. The expression levels of *dsx-A* **(A)** and *dsx-a* **(B)** in the hindwing and *dsx-A* **(C)** and *dsx-a* **(D)** in the abdomen were estimated by reverse transcription quantitative PCR (RT-qPCR) using *RpL3* as an internal control. **(E–G)** Gene expression levels in mimetic (*dsx* genotype: *Hh*) and non-mimetic (*dsx* genotype: *hh*) *Papilio polytes* females and males (*dsx* genotype: *Hh*) at P2, P5, and P10. The wing and abdominal color patterns of mimetic and non-mimetic females. *dsx-H* **(E)** and *dsx-h* **(F)** expression levels in the abdomen were estimated by RT-qPCR using *RpL3* as an internal control. **(A–F)** Yellow, gray, and black bars show the expression levels of mimetic females, non-mimetic females, and males, respectively. Error bars represent standard errors. Different letters indicate significant differences (Tukey’s *post hoc* test, P<0.05).

We examined the expression pattern of *dsx* in *P*. *memnon* hindwings and abdomen (A5 segment) at P2, P5, and P10 using RT-qPCR. First, mimetic (*Aa*) females had high *dsx-A* expression in their hindwings at P2. Males had significantly less *dsx-A* expression than mimetic females at P2 and tended to have reduced expression at P5 and P10 (**Figure 2A**). In addition, *dsx-a* was less expressed in the hindwings of mimetic and non-mimetic females and males at P2 and P5, but expression significantly increased at P10 (**Figure 2B**). *P. polytes* also show a pattern of high expression of mimetic *dsx* (*dsx-H*) in the early pupal stage and high expression of non-mimetic *dsx* (*dsx-h*) in the late pupal stage, suggesting that *cis-*elements or other factors (20) may differentially regulate expression.

Next, we examined the expression pattern of *dsx-A* and *dsx-a* in the abdomen. The results showed that *dsx-A* expression was not statistically significant in mimetic females but tended to be highly expressed at P2 (**Figure 2C**). In the abdomen, unlike in the hindwings, *dsx-A* was expressed similarly in males at P2 and mimetic females (**Figure 2C**). Some P10 males also showed high expression of *dsx-A*, with significant individual variation (**Figure 2C**). *dsx-a* expression in the abdomen differed from the pattern of *dsx-a* expression in the hindwing, with significantly higher expression at P2 (**Figure 2D**). In the abdomen, as in the hindwing, there was no significant difference in *dsx-a* expression among mimetic females, non-mimetic females, and males (**Figures 2B** **, D**). In addition, *dsx-A* and *dsx-a* expression levels relative to the *RpL3* internal standard were considerably higher in the abdomen than in the hindwing, and there was no contrasting expression pattern between *dsx-A* and *dsx-a* as observed in the hindwing (**Figures 2A** **–D**). There may be different mechanisms regulating *dsx* expression in the hindwing and abdomen.

In closely related *P*. *polytes*, no mimetic traits were observed in the abdomen, even in the mimetic females (**Figure 2G**). For comparison, we examined *dsx-H* and *dsx-h* expression levels in the abdomens of *P*. *polytes*. The results showed that *dsx-H* expression tended to be higher in the mimetic females at P2 and lower in the males (**Figure 2E**). *dsx-h* expression was also low in males at P2, P5, and P10 but was consistently expressed in mimetic and non-mimetic females, with a trend toward greater expression at P10, although this was not statistically significant (**Figure 2F**). The absence of mimetic traits in *P*. *polytes* mimetic female abdomens may not be due to the lack of *dsx* expression in the abdomen but rather to differences in *dsx* function between the two species. Comparing the amino acid sequences of DSX-A/H and DSX-a/h in *P*. *polytes* and *P*. *memnon* revealed no common amino acid substitutions between alleles (8).

**Figure 3:**
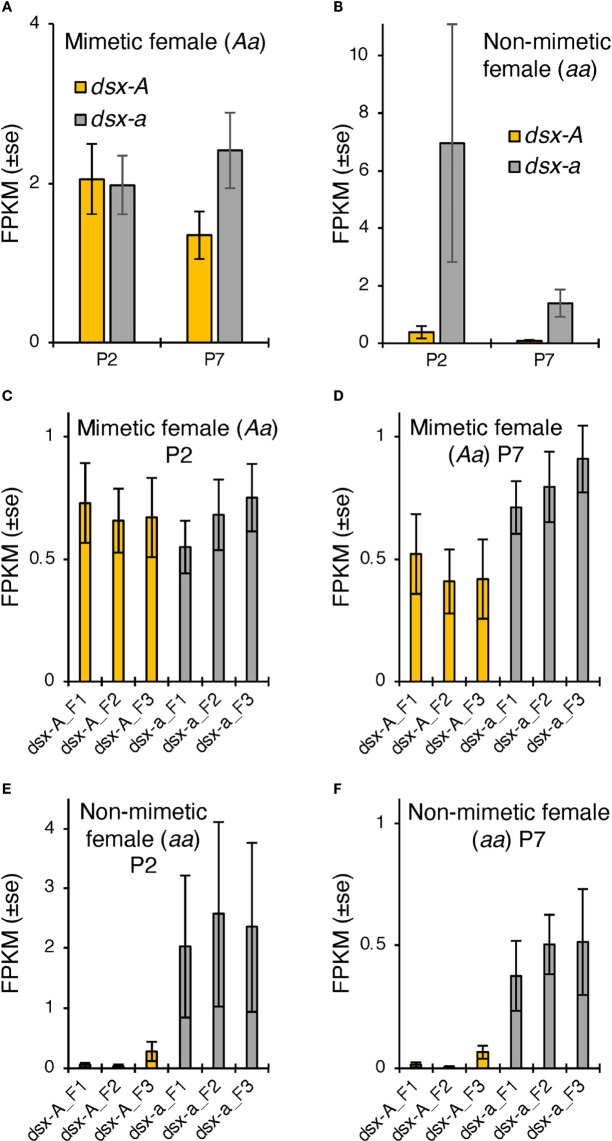
**(A, B)** *dsx-A* and *dsx-a* expression levels in *Papilio memnon* hindwings of mimetic (*dsx* genotype: *Aa*; **(A)** and non-mimetic (*dsx* genotype: *aa*; **(B)** females at two (P2) and seven days after pupation (P7). The mean fragment per kilobase of transcript per million mapped reads (FPKM) values by RNA sequencing (RNA-seq) are shown with SE. Different letters indicate significant differences (Tukey’s *post hoc* test, P<0.05). Yellow and gray bars show the *dsx-A* and *dsx-a* expression levels, respectively. **(C–F)** Expression levels of each *dsx* isoform in *P. memnon* mimetic (*dsx* genotype: *Aa*; **(C, D)** and non-mimetic (*dsx* genotype: *aa*; **(E, F)** females. The mean FPKM values by RNA-seq at P2 and P7 are **(C, E)** and **(D, F)**, respectively. Orange bars indicate the expression levels of *dsx* isoforms from the mimetic **(*A*)** allele, and gray bars indicate the non-mimetic *a* allele. F1, F2, and F3 represent the female isoforms 1, 2, and 3, respectively. There were no statistically significant differences between the isoforms.

We further examined the differences in *dsx-A* and *dsx-a* expression using RNA-seq read data in the hindwings of mimetic females and found no significant expression differences at either P2 or P7. However, *dsx-a* had a higher expression at P7 (**Figure 3A**). The trends of high *dsx-a* expression in the late pupal stage and *dsx-A* being expressed at P2 to a greater extent than at P7 were consistent with the RT-qPCR results. However, the absolute expression of *dsx-A* and *dsx-a* at P2 seemed to be almost the same, and both may be involved in wing color pattern formation. RNA-seq of non-mimetic female hindwings showed a trend toward greater *dsx-a* expression in P2, but this was not statistically significant (**Figure 3B**). This may be because *dsx-a* expression at P2 was highly variable (the expression was considerably greater in only one individual).

We also examined the expression levels of *UXT*, an HDR gene, in P2 and P7 using RNA-seq read data. The results showed that expression was high at P2 in both mimetic and non-mimetic females, and there was no significant difference between *UXT* from the *A* (*UXT-A*) and *a* alleles (*UXT-a*) (**Figure S2**). This trend was consistent with the expression pattern of *UXT* in *P*. *polytes* (20). In addition, no expression in the hindwings has been confirmed for *Nach-like*, which is present within HDRs (8).

Finally, we examined the expression levels of the three isoforms in *dsx* females based on RNA-seq read data and found no significant differences in expression between isoforms in either P2 or P7 mimetic or non-mimetic females (**Figures 3C** **–F**). This isoform expression pattern is similar to that of *P*. *polytes* (20).

### Functional Analysis of *dsx* by Electroporation-Mediated RNAi

**Figure 4:**
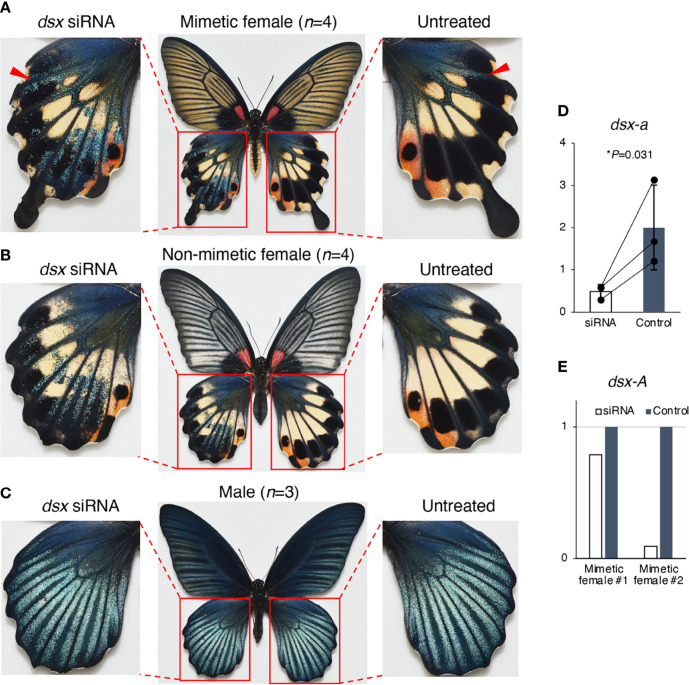
*dsx* knockdown in the hindwings of *Papilio memnon* mimetic **(A)** and non-mimetic **(B)** females and males **(C)**. Small interfering RNA (siRNA) targeted the sequence common to *dsx-A* and *dsx-a*, and all the female and male isoforms (*dsx*-common siRNA) were injected into the left pupal hindwing immediately after pupation and electroporated into the dorsal side. *dsx* knockdown changed the mimetic and non-mimetic female color patterns to resemble the male pattern **(A, B)**, but no phenotypic change was observed in males **(C)**. Pale yellow and red spots disappeared in the knockdown side of mimetic and non-mimetic females, resulting in a phenotype with blue scales on a black background **(A, B)**. The red arrowheads indicate the area that originally had blue scales (untreated side) and where the knockdown appears to have expanded the area of blue scales (*dsx* siRNA side). **Supplementary Figure S3** shows the other replicates. **(D, E)** *dsx-a* and *dsx-A* gene expression levels in the knockdown wings two days after pupation. When *dsx* was knocked down by *dsx*-common siRNA, *dsx-a* was significantly downregulated **(D)**, and *dsx-A* was downregulated in all two individuals tested **(E)**. White and gray bars show the expression in treated and untreated hindwings, respectively. We estimated the gene expression levels by reverse transcription quantitative PCR (RT-qPCR) using *RpL3* as the internal control. **(D)** Error bars show the standard deviation of three biological replicates. *P<0.05 for Student’s *t*-test. **(E)** The expression levels are shown as relative values, with the expression level in the untreated wing taken as 1.

We conducted RNAi using *in vivo* electroporation with three different siRNAs to investigate the function of *dsx* in the hindwing and abdomen of *P*. *memnon* (**Figure 1C**). First, the pale yellow and red spots in the center and outer margin of the hindwings disappeared, and the phenotype of blue scales on a black background, as seen in males, was observed in the hindwings of both mimetic and non-mimetic females introduced with siRNAs targeting *dsx-common* siRNA (**Figures 4A** **, B**, **Figure S3**). This blue scale was originally seen in small amounts in both mimetic and non-mimetic females, and some areas with blue scales on the control side appeared to spread on the knockdown side (indicated by arrowheads in **Figure 4A**). The blue scales also varied among individuals, with some females having no blue scales at all while others initially had many (**Figure S6C**). The variation in the blue scales may be due to variations in *dsx* expression among parts of the hindwing and individuals. In contrast, there were no distinct changes in males after knockdown with *dsx-common* siRNA (**Figure 4C**, **Figure S3**). *dsx-a* and *dsx-A* expression levels were examined by RT-qPCR on the *dsx-common* siRNA-introduced side and the untreated side of the hindwing. *dsx-a* was significantly downregulated on the siRNA-introduced side, and *dsx-A* was downregulated on the siRNA-introduced side in both individuals examined (**Figures 4D** **, E**).

**Figure 5:**
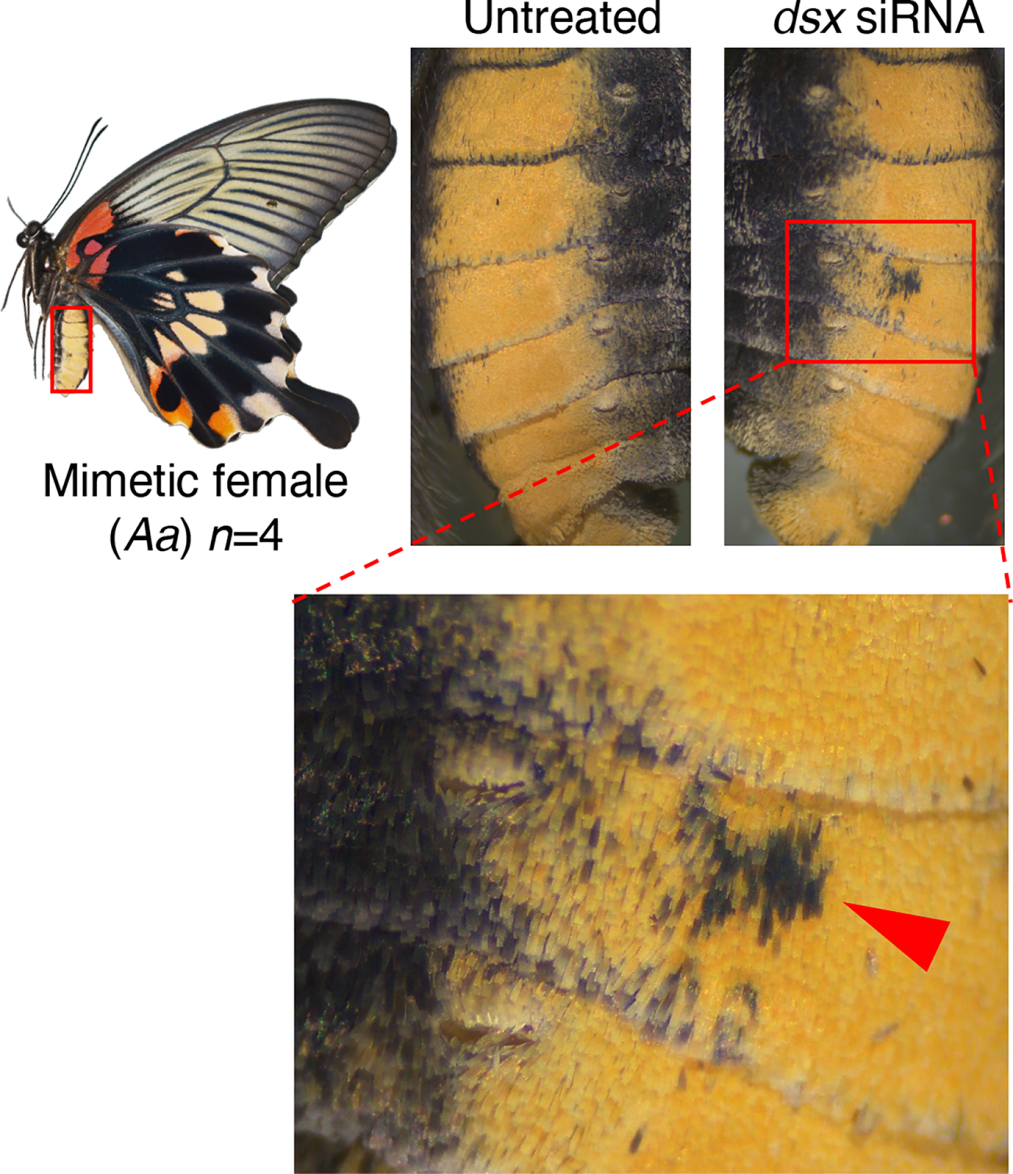
*dsx* knockdown in the abdomen of *Papilio memnon* mimetic females. Small interfering RNA (siRNA) targeted the sequences common to *dsx-A* and *dsx-a*, and all the female and male isoforms (*dsx*-common siRNA) were injected into the abdomen during the wandering stage of final instar larvae and electroporated into the fifth abdominal segment. Knockdown changed the yellow scales characteristic of mimetic forms to the black scales seen in non-mimetic forms and males. The red arrowheads indicate the changed area. **Supplementary Figures S4** and **S5** show the other replicates.

Next, when *dsx-common* siRNA was introduced into the abdomen for knockdown, the yellow scales characteristic of mimetic females changed to the black scales seen in non-mimetic females and males in the area where the siRNA was introduced (**Figures 5**, **Figure S4**). Conversely, no phenotypic change was observed in the abdomens of non-mimetic females or males due to knockdown (**Figure S5**). Scanning electron microscope (SEM) observations revealed no significant differences in the ultrastructure of the yellow and black scales of *P*. *memnon* and *P*. *polytes* abdomens (**Figure S8**), suggesting that the pigment synthesis system, rather than the three-dimensional structure of the scales, was altered by RNAi. These results indicate that *dsx* suppresses the formation of male blue scales and induces the formation of female-specific pale yellow or red spots in the hindwing, whereas in the abdomen, *dsx* switches from black to yellow scales. Thus, *dsx* is involved in the phenotypic switch from male to mimetic and non-mimetic females in the hindwing and abdomen alike, but the detailed mechanisms, such as differences in the downstream target genes it regulates, may be different. When *dsx-F* siRNAs were introduced into the hindwings of mimetic females, we observed the same changes as when *dsx-common* siRNA was introduced (**Figure S6**), reconfirming the function of *dsx* in the hindwing. In this study, siRNA was introduced into the dorsal side of the hindwing to observe phenotypic changes caused by knockdown, whereas *dsx-F* siRNA was introduced into the ventral side of the mimetic female as well. The pale yellow and red spots in mimetic females disappeared when *dsx-F* siRNA was introduced into the ventral side, and a blackish color similar to that seen in males appeared (**Figures S6D, E**). Unlike the dorsal side, blue scales were barely visible on the male ventral sides (**Figure S3J**), and blue scales did not appear when knockdown was performed on the ventral side of mimetic females (**Figures S6D, E**).

**Figure 6:**
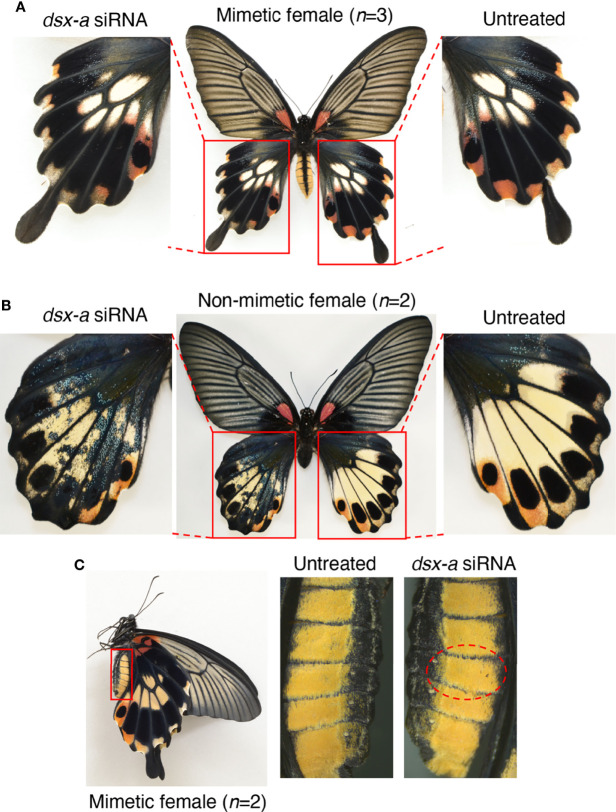
*dsx-a* knockdown in the hindwings of *Papilio memnon* mimetic **(A)** and non-mimetic **(B)** females, and the abdomen of mimetic females **(C)**. Small interfering RNAs (siRNAs) targeting *dsx-a* (*dsx-a* siRNA) were injected into the left pupal hindwing immediately after pupation and electroporated into the dorsal side **(A, B)** and the abdomen during the wandering stage of the final instar larvae and electroporated into the fifth abdominal segment **(C)**. No distinct phenotypic change was observed in mimetic females **(A)**, but *dsx-a* knockdown changed the non-mimetic female color pattern to resemble the male pattern **(B)**. As in the knockdown by *dsx*-common siRNA, pale yellow and red spots disappeared in the knockdown side of non-mimetic females, resulting in a phenotype with blue scales on a black background **(B)**. **(C)** In the knockdown of the fifth abdominal segment in mimetic females, no phenotypic change was observed. Red dotted circles show the electroporated area. **Supplementary Figure S7** shows the other replicates.

Finally, to investigate the role of *dsx-A* and *dsx-a*, we performed a knockdown by introducing *dsx-a* siRNA. When *dsx-a* siRNAs were introduced into the hindwings, we observed no distinct changes in mimetic females (**Figure 6A**, **Figure S7**). However, in the knockdown region of non-mimetic females, the pale yellow and red spots disappeared as they did when *dsx-common* siRNAs were introduced, resulting in a blue-scale phenotype on a black background, the phenotype seen in males (**Figure 6B**, **Figure S7**). Since the same phenotypic changes were observed when *dsx-common* siRNA was introduced only in non-mimetic females, it suggested that only *dsx-a* could be specifically knocked down, although this was not confirmed directly by RT-qPCR. Finally, no phenotypic changes were observed when *dsx-a* siRNA was introduced into the abdomen (**Figure 6C**, **Figure S7**). *dsx-a* siRNA did not cause phenotypic changes in the hindwings or abdomen of mimetic females, as observed when *dsx-common* siRNA was introduced, suggesting that *dsx-A* is critical in the formation of hindwing and abdominal mimicry traits.

## Discussion

For the first time, our functional analysis results show that *dsx* is necessary for controlling hindwing color pattern and abdominal yellow in the *P. memnon* mimicry polymorphism. We also showed that *dsx-a* determines the color pattern of the non-mimetic hindwing, suggesting that *dsx-A* is involved in regulating the mimetic hindwing color pattern and abdominal yellow. There were no amino acid substitutions inside the two important domains in *dsx-A and dsx-a* (DNA binding and dimerization domains), and there were only four amino acid substitutions in the entire ORF (8). Therefore, there may be no difference in protein function between DSX-A and DSX-a, and mimetic and non-mimetic polymorphisms may be controlled by differences in *dsx-A* and *dsx-a* expression regulation. We found a difference in the expression pattern between *dsx-A* and *dsx-a* in the hindwing (*dsx-a* was highly expressed in the late pupal stage; **Figure 2B**). However, the absolute expression of *dsx-A* and *dsx-a* was not significantly different in the early pupal stage (P2), which is considered important for mimetic trait formation (**Figure 3A**). Although *dsx-A* is important for the formation of mimetic phenotype, whether there is a difference in protein function between DSX-*A* and DSX-*a*, or whether the *dsx* expression pattern is important, remains to be elucidated and requires further studies, including *dsx-A* specific knockdown.

Discrepancies in the expression patterns between *dsx-A* and *dsx-a* indicate that there may also be differences in expression regulation by *cis-*elements and other factors. In particular, *dsx-A* expression may be suppressed in the hindwing of the second half of pupae compared to *dsx-a*, suggesting that *dsx-A* and *dsx-a* expression patterns may be regulated separately. The color pattern in the wing in the late pupal stage has already been determined, and expression regulation in the late stage may not be related to color pattern formation in the wing. However, *dsx* may be involved, for example, in the formation of some phenotypes other than color pattern, or conversely, have toxic effects. In *P*. *polytes*, while *dsx-H* has been reported to have deleterious effects on longevity and reproduction (53), no deleterious effects of *dsx-A* have been reported to date in *P*. *memnon* (49), and the role of *dsx* in the later stage of pupal wing is unknown.

Furthermore, comparing *P*. *polytes* and *P*. *memnon*, *dsx-H* is important for mimetic female wing pattern formation in *P*. *polytes*. However, there are no common amino acid substitutions between *P*. *polytes* and *P*. *memnon*, and the amino acid substitutions that are important for the formation of mimetic traits are unknown. Therefore, even if DSX-A/H and DSX-a/h have different functions as proteins, the amino acid sequences that characterize the function of each protein are different between *P*. *polytes* and *P*. *memnon*. However, these amino acid mutations are unknown at present. In the future, we hope to clarify the functions of DSX-A and DSX-a by studying the interaction between DSX, a transcription factor, and DNA using chromatin immunoprecipitation sequencing (ChIP-Seq) and other methods.

**Figure 7:**
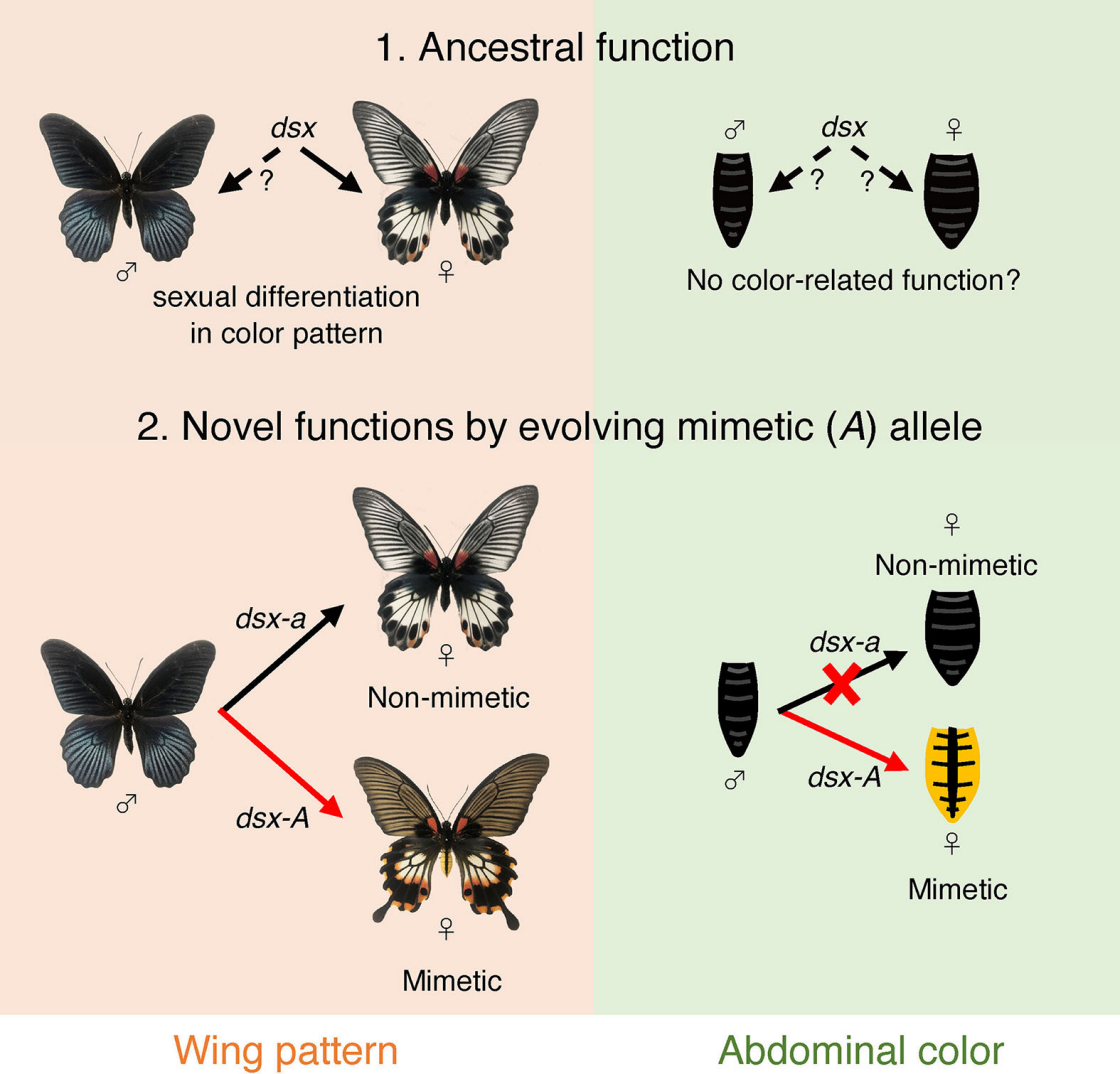
Model diagram of the evolution of mimetic *dsx* (*dsx-A*) and the acquisition of mimetic traits. Originally, *dsx* may have helped control sexual dimorphism in the hindwing, but its ancestral function concerning abdominal coloration is unknown, and it may not have been involved in determining abdominal coloration (1. Ancestral function). In *P*. *memenon*, the function of *dsx* in the male wing is not known, and we do not know if the *dsx* male isoform had any ancestral function in the wing. Thus, the male wing pattern may be a default and ancestral trait, and only female isoform may work to form sexual dimorphism in the wing. In the hindwing, *dsx-A* differentiation has resulted in the appearance of the mimetic form only in females, and ancestral *dsx* (i.e., *dsx-a*) is involved in phenotyping the non-mimetic form (2. Novel functions by evolving mimetic (*A*) allele). On the other hand, only *dsx-A* appears to be involved in abdominal coloration, while *dsx-a* appears to have no function.

Notably, *dsx-A* controls two mimetic traits: hindwing color pattern and yellow abdomens. It is thought that the female-limited polymorphism originally evolved from sexual dimorphism and that female-limited dimorphism evolved by co-opting *dsx*, which originally controlled the differences between male and female wing patterns (**Figure 7**) (14, 17). On the other hand, for the abdomen, there is no polymorphism between males and non-mimetic females in *P*. *memnon*, and they have the same black coloration. Therefore, it is unlikely that there was originally sexual dimorphism of the abdomen and that the function of *dsx*, which controls sexual dimorphism of the abdomen, was co-opted to control the yellow abdomen that is unique to mimetic females. Few species of the genus *Papilio* are known to have abdominal colors that differ between males and females (although *P*. *aegeus*, *P*. *jordani*, and *P*. *gulaucus* have polymorphic abdomens. See Introduction.). How *dsx* functions in the abdomen is unknown. In *Drosophila melanogaster*, the abdomen is melanized only in males. Abdominal sexual dimorphism in *D. melanogaster* is regulated by the female isoforms of *dsx* and *Abdominal-B* promoting the expression of the transcriptional regulator *bric a brac* (*bab*), and by the *dsx* male isoform and *Abdominal-B* repressing *bab* expression (54, 55). Thus, in *P*. *memnon*, *dsx-A* may be involved in forming the yellow abdomen characteristic of the mimetic female by modifying the gene network involved in melanin synthesis in the abdomen. Therefore, *dsx-A* may regulate the expression of different downstream genes in the hindwing and abdomen, which is surprising, given the evolutionary process. Multiple traits of Batesian mimicry are considered to have no mimetic effect unless they are all set. For example, if the hindwings have ancestral (non-mimetic) traits but only the abdomen has mimetic traits, mimicry will not function, and conversely, the species will stand out to predators, making it more susceptible to predation. Multiple mimetic traits in the hindwing and abdomen must evolve concurrently. However, if the downstream genes regulated by *dsx* are different in the hindwing and abdomen, it would be difficult to acquire two mimetic traits simultaneously.

The model species for *P*. *memnon* includes *P. aristolochiae*, *A. coon*, and *A. polyeuctes*, but most of the model species have red abdomens, although some have yellow abdomens. Therefore, the abdomen of the *P*. *memnon* mimetic female may be an imperfect mimicry that failed to acquire red coloration and acquired yellow as close to it as possible as a mimetic trait (56, 57). On the other hand, *P*. *polytes* also mimic *P. aristolochiae*, but they do not have any mimetic traits in their abdomen (i.e., there is no difference between mimetic and non-mimetic types of *P*. *polytes*; **Figure 2G**). Thus, abdominal color may not be an important mimetic trait. Therefore, in *P*. *memnon*, the hindwing mimetic trait may have evolved first, and the abdominal mimetic trait may have been acquired later. If this occurred, a mutation in the regulatory region of the downstream gene, whose expression is controlled by *dsx* in the abdomen, may have allowed only *dsx-A* to form the mimetic trait. Further studies on the genes downstream of *dsx-A* in the hindwing and abdomen are needed.

Another mimetic trait found in *P*. *memnon* is the tail of its hindwings. So far, whether *dsx* is involved in controlling the presence or absence of tails has not been investigated. The boundary lacuna is already formed, and the wing shape is determined in the early fifth instar larvae in lepidopterans. In *P*. *polytes* and *P*. *memnon*, the shape of the tails can be seen in the wing discs of fifth instar larvae, suggesting that the presence or absence of tails was determined before this stage. Functionally analyzing the genes involved in the tails is difficult because we performed the functional analysis using RNAi by electroporation by introducing siRNA immediately after pupation to knockdown the genes.

*dsx* was involved in the formation of mimetic traits in at least two separate locations: the color pattern of the hindwings and the yellow color of the abdomen. However, the differences in expression patterns between the hindwing and abdomen suggest that diverse genetic elements may be involved in molecular mechanisms, such as regulating *dsx* expression. In *P*. *polytes*, it has been suggested that a long noncoding RNA, *U3X*, regulates *dsx* and *UXT* expression in the HDR (20). Although *U3X* has not been found in *P*. *memnon*, enhancers or *cis-*elements within the HDR may regulate the mimicry phenotype, a set of multiple traits, by regulating *dsx* expression. In other words, it is not just a single gene (*dsx*) that regulates mimicry, but the HDR may still function as a supergene. Further studies on the function of *UXT* and the regulatory regions in HDRs will reveal the regulatory mechanism of Batesian mimicry in *P*. *memnon* by the supergene.

## Data Availability Statement

The datasets presented in this study can be found in online repositories. The names of the repository/repositories and accession number(s) can be found below: https://www.ncbi.nlm.nih.gov/bioproject/851275. Any further queries should be directed to the corresponding author.

## Author Contributions

SK and HF conceived and designed the study. SK and C-PL collected butterflies in Taiwan. SK conducted experiments. SK and HF wrote the paper. HF supervised this project. All authors reviewed the manuscript.

## Funding

This work was supported by Ministry of Education, Culture, Sports, Science and Technology/Japan Society for the Promotion of Science KAKENHI (22128005, 15H05778, 18H04880, 20H04918, 20H00474 to HF; 19J00715 to SK).

## Conflict of Interest

The authors declare that the research was conducted in the absence of any commercial or financial relationships that could be construed as a potential conflict of interest.

## Publisher’s Note

All claims expressed in this article are solely those of the authors and do not necessarily represent those of their affiliated organizations, or those of the publisher, the editors and the reviewers. Any product that may be evaluated in this article, or claim that may be made by its manufacturer, is not guaranteed or endorsed by the publisher.
