# Supplementary material for: doublesex Controls Both Hindwing and Abdominal Mimicry Traits in the Female-Limited Batesian Mimicry of Papilio memnon
Source: Front Insect Sci. 2022 Jul 12;2:929518. doi: 10.3389/finsc.2022.929518 (PMC10926503; doi:10.3389/finsc.2022.929518)
Supplement: Supplementary file 1 [file DataSheet_1.pdf]

Supplementary Material

1 Supplementary Figures and Tables

1.1 Supplementary Figures

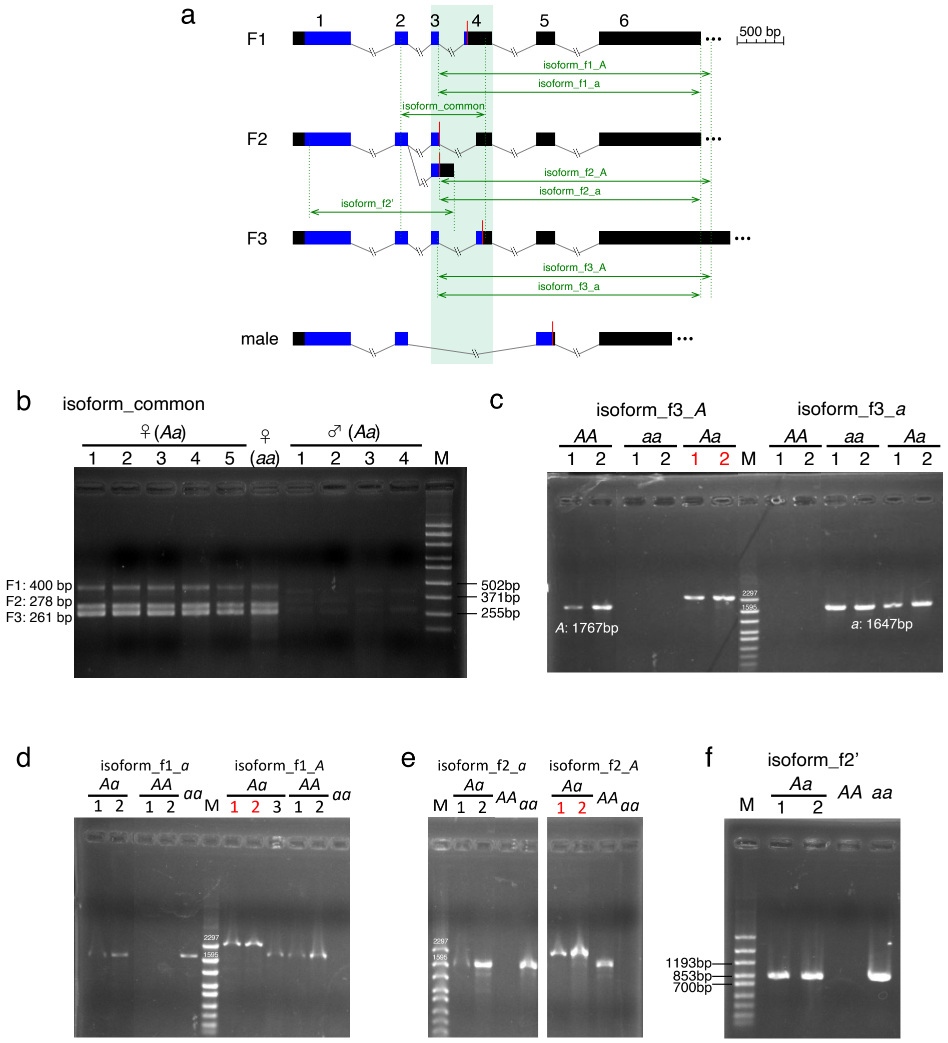

Supplementary Figure S1. Identification and summary of *dsx* isoforms in *Papilio memnon*. First, RNA sequencing (RNA-seq) read data was used to obtain three major *dsx* isoform types in females and one in males (a). However, no sample contained all isoforms, and the isoforms obtained were unevenly distributed among individuals (Table S2). Hence, PCR was used to reconfirm the three female isoforms (b). The length of the sequence obtained by each isoform was different for exon 6. For this, we designed a primer based on female isoform 3, for which the longest sequence was obtained by RNA-seq, and found that exon 6 was at least 1200 bp long in all isoforms (c–e). (b) PCR was performed on the isoform-common region shown in (a). This resulted in three PCR bands using hindwing samples from five *Aa* females and one *aa* female on the second day after pupation (P2), each with the lengths expected from female isoforms 1, 2, and 3 (F1, F2, and F3) (F1: 400 bp, F2:

278 bp, F3: 261 bp). No PCR bands were identified in the four *Aa* males. The following primers were used for PCR: Pme_dsx_isoform_f_common (Forward: ATGATGCCGTTAGTCCTGGT; Reverse: CCACACAAAACACAGCACTTG). (c) PCR was performed on the hindwing of a P2 female to amplify the isoform_f3_A and isoform_f3_a regions shown in (a). As expected, we obtained 1767 bp and 1647 bp bands for *A* and *a,* respectively. Exon 6 of F3 contained about 1200 bp of sequence. On the other hand, PCR with two *Aa* females (shown in red) yielded a longer band than expected. The following primers were used for PCR: Pme_dsx_isoform_f3_AA (Forward: ACTCGACACGCCAGAAAATG; Reverse: TCCCTGCACTGTTCGATTCA), Pme_dsx_isoform_f3_aa (Forward: ACTCGACACGCCAGAAAATG; Reverse: TTTACTGGCCACTAACGGGGT). (d) PCR was performed on the hindwing of P2 females to amplify isoform_f1_A and isoform_f1_a regions shown in (a). The 3' side of this region (reverse primer) is not included in the contig obtained by RNA-seq. However, as with F3, exon 6 was thought to be connected by a longer sequence than exon 6 in the contig obtained by RNA-seq. Hence, we used isoform_f3_A and isoform_f3_a reverse primers. As a result, a band of the expected length was obtained. Similar to (c), a band longer than expected was obtained in two *Aa* females (red). PCR was performed using the forward primer common to F1, Pme_dsx_isoform_f1 (forward: GAACTCGACACGCCATGACC) and Pme_dsx_isoform_f3. AA and Pme_dsx_isoform_f3_aa reverse primers were used. (e) PCR was performed to amplify isoform_f2_A and isoform_f2_a in (a) using the forward primer Pme_dsx_isoform_f2 (Forward: GAACTCGACACGCCAGTATG) and reverse primers Pme_dsx_isoform_f3_AA and Pme_dsx_isoform_f3_aa. (f) PCR was performed using the following primers to amplify isoform_f2' in (a), and the results were as expected: Pme_dsx_isoform_f2prime (Forward: TCTCCCGACGAATGTGACG; Reverse: ACGAGGTGGCACTAAACTTG), product size 871 bp.

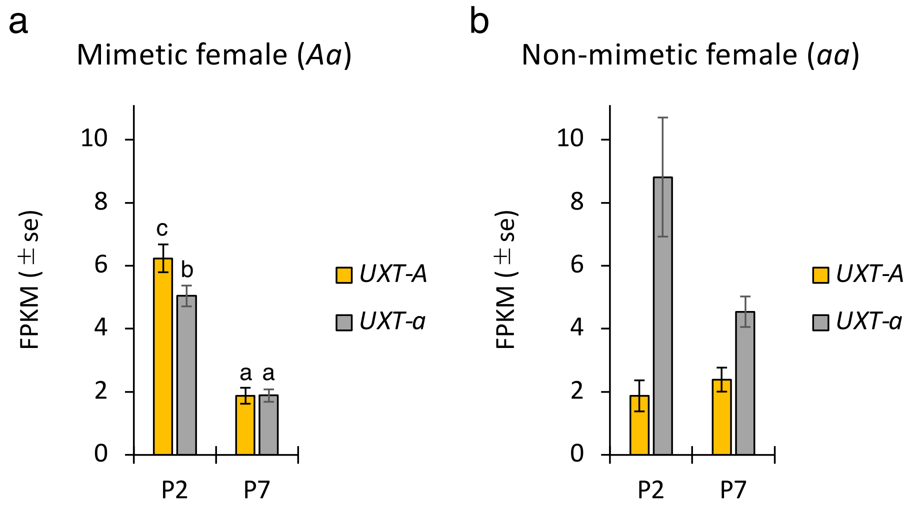

Supplementary Figure S2. Expression levels of *UXT* from the A (*UXT-A)* and a (*UXT-a)* alleles in the hindwings of mimetic (*dsx* genotype: *Aa*; a) and non-mimetic (*dsx* genotype: *aa*; b) females two (P2) and seven days after pupation (P7) in *Papilio memnon*. The mean fragment per kilobase of transcript per million mapped reads (FPKM) values by RNA sequencing (RNA-seq) are shown with SE. Different letters indicate significant differences (Tukey's post hoc test, P<0.05). Yellow and gray bars show the expression levels of *UXT-A* and *UXT-a*, respectively.

(Continue to the next page)

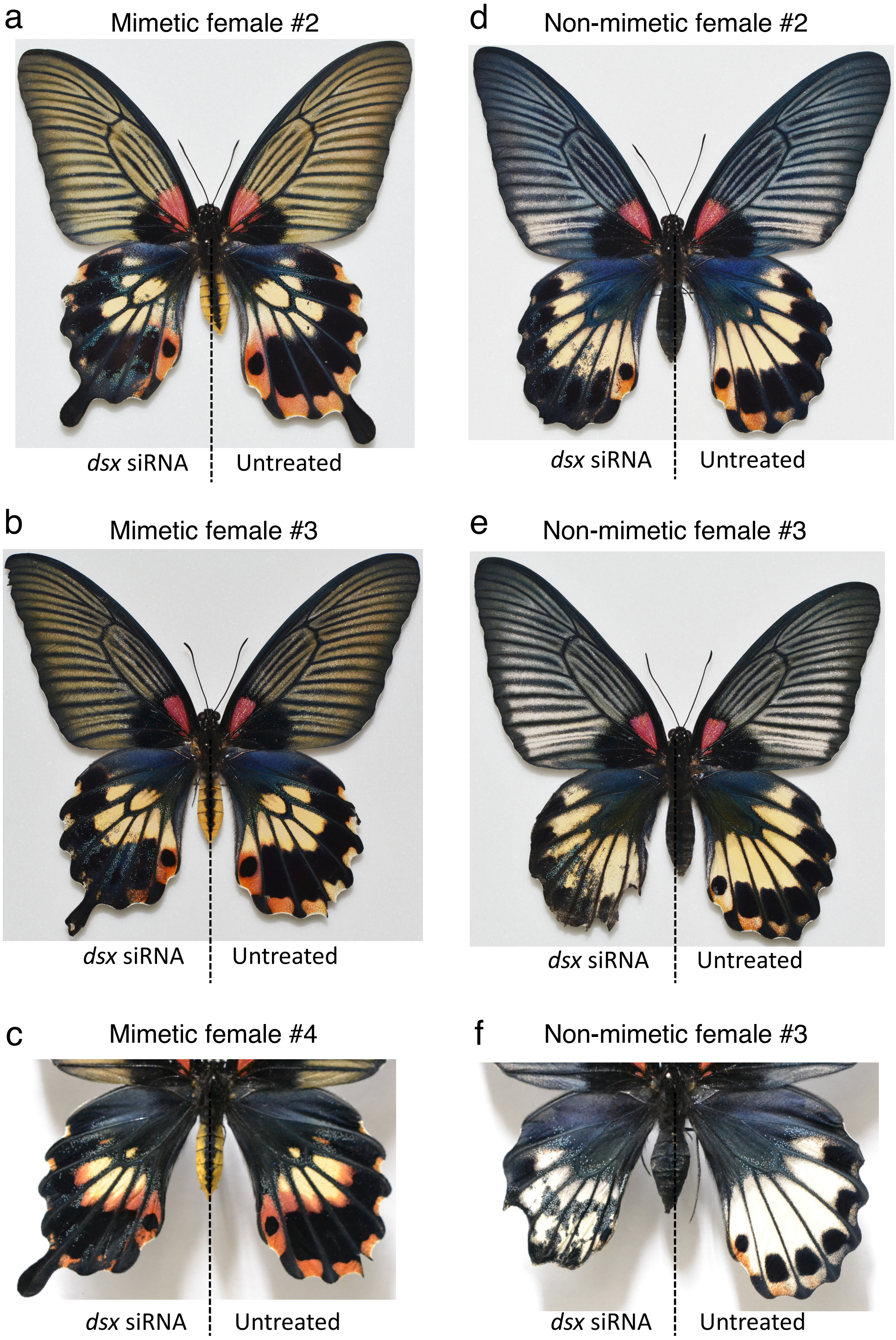

Supplementary Figure S3. *dsx* knockdown in the hindwings of mimetic (a–c) and non-mimetic (d–f) females and males (g–j). *dsx-common* siRNAs knocked down *dsx*. Other replicates of Figure 4 (k) show the target sequence of *dsx-common* siRNA, common to *dsx-A*, *dsx-a,* and all female and male isoforms.

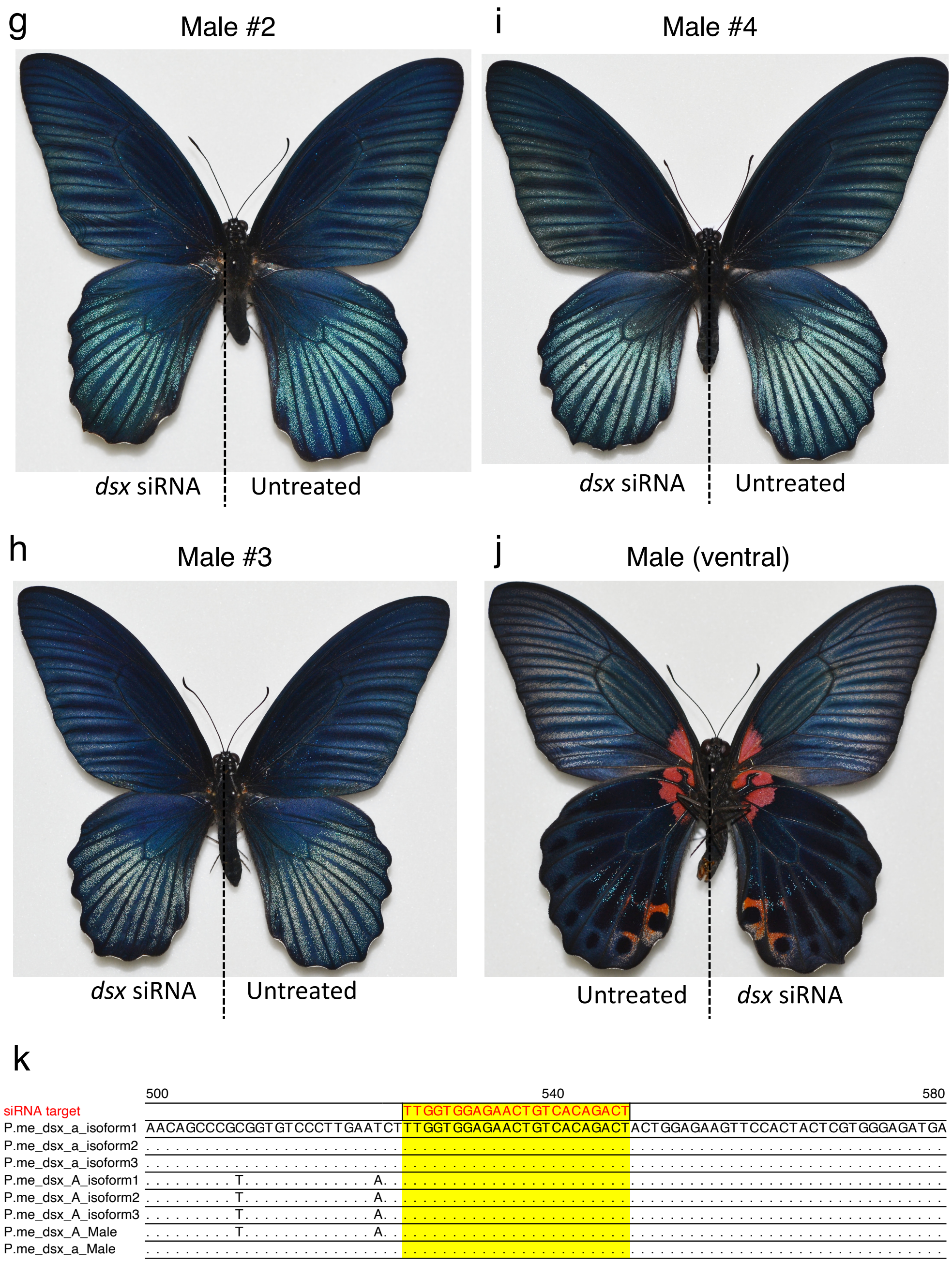

Supplementary Figure S3. *dsx* knockdown in the hindwings of mimetic (a–c) and non-mimetic (d–f) females and males (g–j). *dsx-common* siRNAs knocked down *dsx*. Other replicates of Figure 4 (k) show the target sequence of *dsx-common* siRNA, common to *dsx-A*, *dsx-a,* and all female and male isoforms.

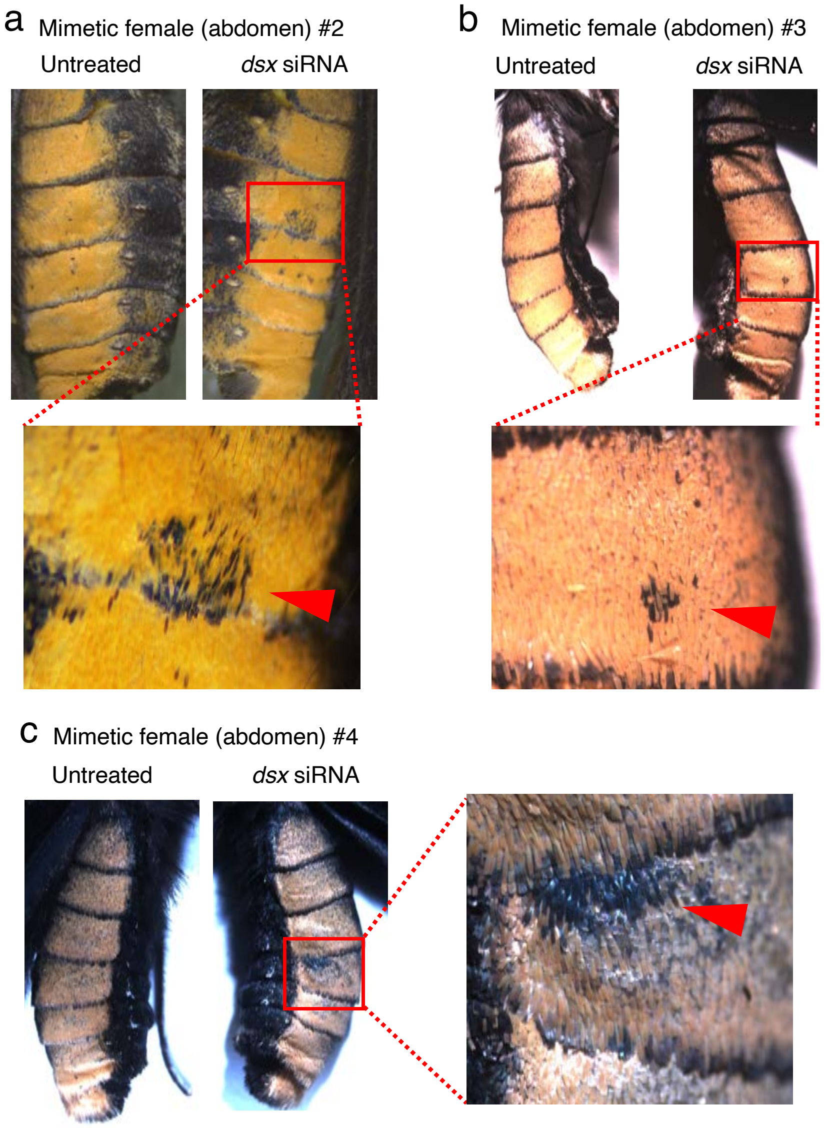

Supplementary Figure S4. *dsx* knockdown in the abdomen of *Papilio memnon* mimetic females. *dsx-common* siRNA knocked down *dsx*. Other replicates of Figure 5. Red arrowheads indicate the area *dsx* knockdown changed.

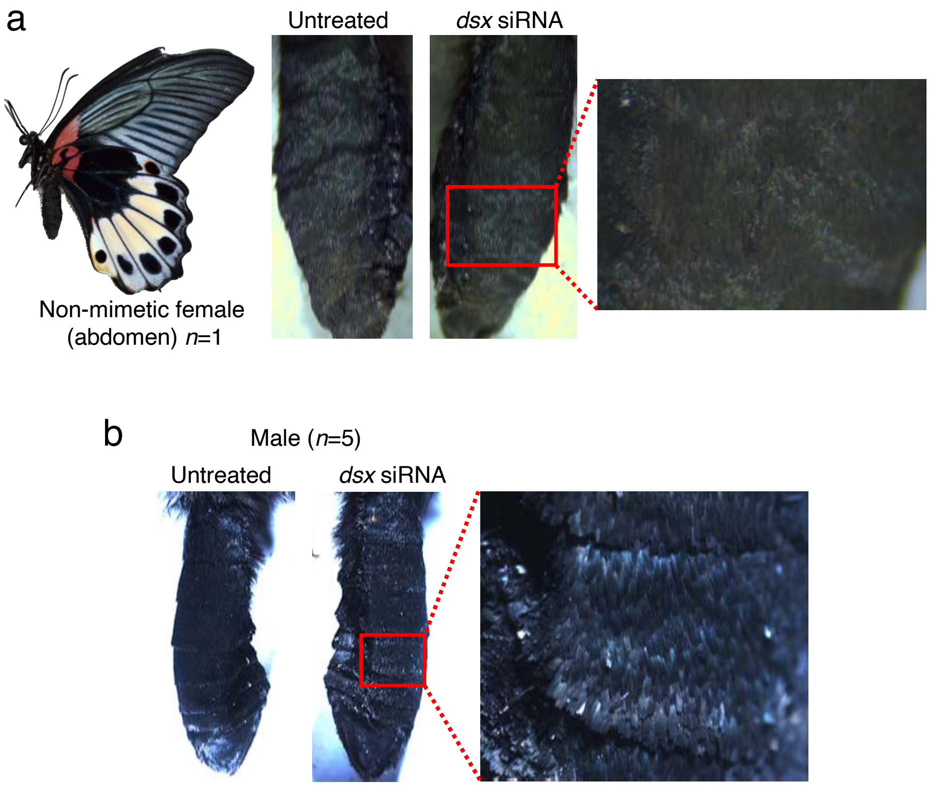

Supplementary Figure S5. *dsx* knockdown in the abdomen of a non-mimetic *Papilio memnon* females and males. *dsx-common* siRNA knocked down *dsx*. No phenotypic change was found by knockdown in the non-mimetic female and males.

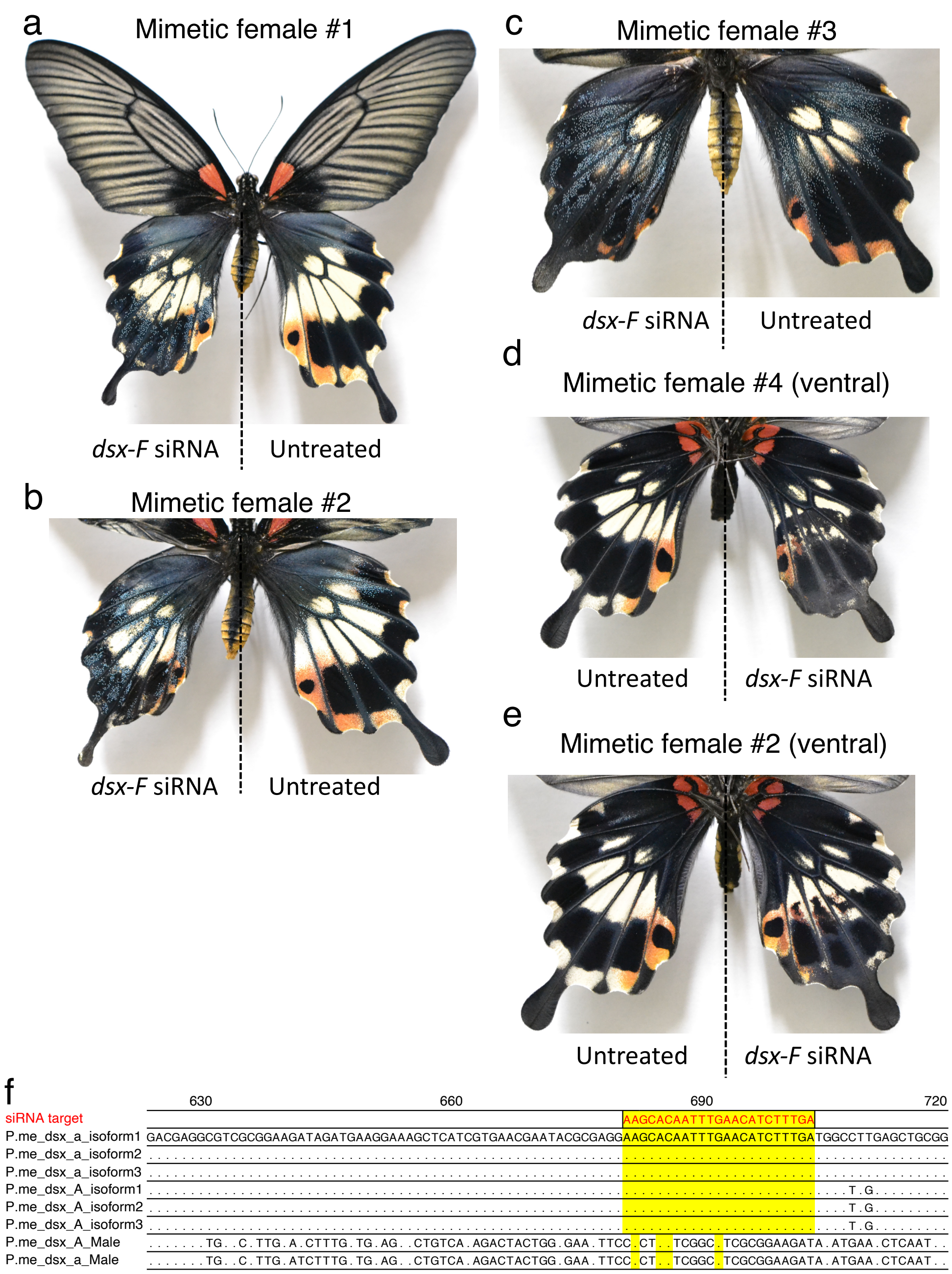

Supplementary Figure S6. *dsx* knockdown in the hindwings of *Papilio memnon* mimetic females. Small interfering RNAs (siRNAs) targeting the sequence specific to all female isoforms (*dsx-F* siRNA) were injected into the left pupal hindwing immediately after pupation and electroporated into the dorsal (a–c) or ventral (d–e) sides. *dsx-F* knockdown changed the mimetic and non-mimetic female color pattern to resemble the male pattern. Pale yellow and red spots disappeared on the knockdown side, resulting in a phenotype with blue scales on a black background on the dorsal side (a–c). On the ventral side, pale yellow and red spots disappeared on the knockdown side, but there was no blue scale as seen in the color pattern of male ventral wings (d, e). (f) Shows the target sequence of *dsx-F* siRNAs, which is common to all female isoforms.

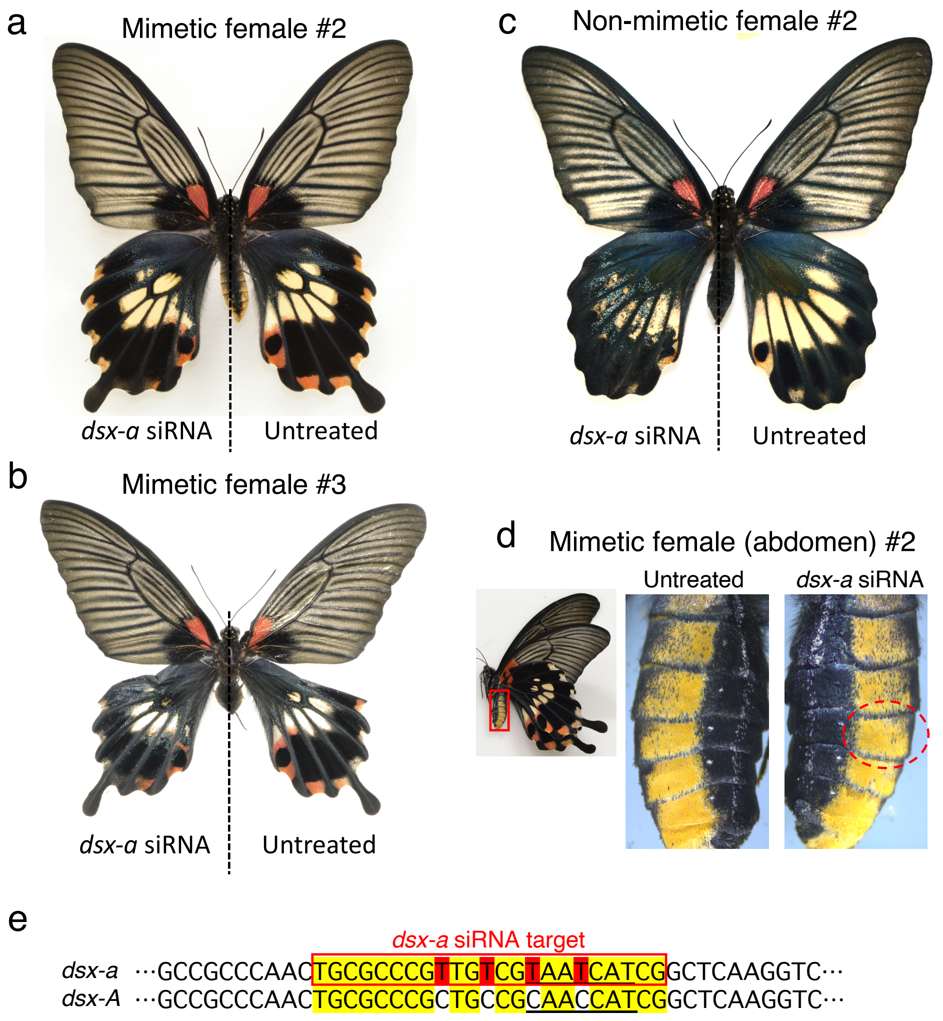

Supplementary Figure S7. *dsx-a* knockdown in the hindwings of mimetic (a, b) and non-mimetic (c) females, and the abdomen of a mimetic female (d). Small interfering RNAs (siRNAs) knocked down *dsx-a*. Other replicates of Figure 6 (e) show the target sequence of *dsx-a* siRNA, which is specific to *dsx-a*.

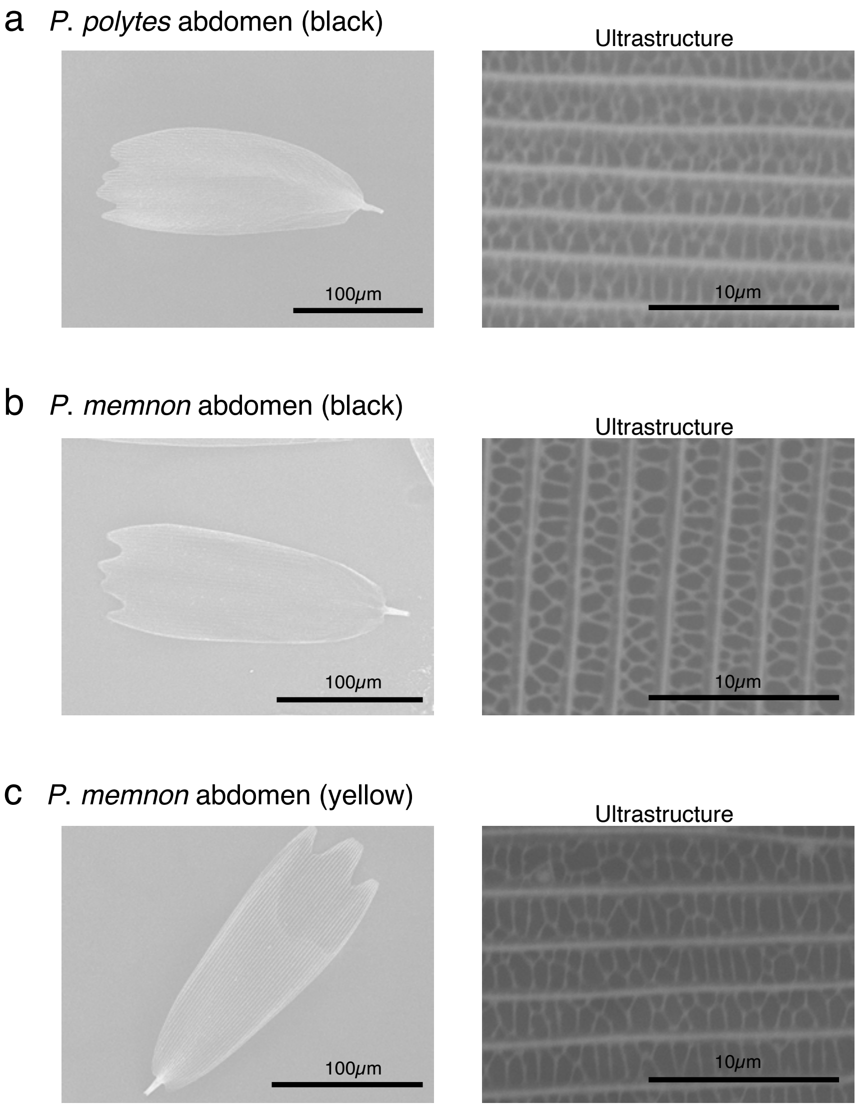

Supplementary Figure S8. Observing the scale structure in the abdomen of *P. polytes* and *P. memnon* using a scanning electron microscope (SEM). SEM observations were made on the abdominal scales (Hitachi Miniscope TM-1000). The differences in the color of the abdomen suggested that there might be differences in the scale structures, but all the observed scales had a fine pore structure. No significant differences were observed between the yellow and black portions of the *P. memnon* abdomen, nor with the *P. polytes* abdomen scales.

1.2 Supplementary Tables

Supplementary Table S1. Sample list of RNA sequencing

Sample ID	sex	genotype	stage	Total size (bp)	Accession number	References
lr860	female	Aa	P7	4,018,429,334	SAMD00052594	Komata et al., 2016
lr861	male	aa	P7	3,966,944,159	SAMD00052595	Komata et al., 2016
mesu6	female	Aa	P7	3,825,155,984	SAMD00074277	Iijima et al., 2018
mesu9	female	Aa	P7	3,475,745,633	SAMD00074278	Iijima et al., 2018
mesu10	female	Aa	P7	3,020,684,426	SAMD00074279	Iijima et al., 2018
osu13	male	Aa	P7	3,560,870,212	SAMD00074280	Iijima et al., 2018
osu20	male	Aa	P7	3,215,735,635	SAMD00074281	Iijima et al., 2018
osu22	male	Aa	P7	3,489,366,132	SAMD00074282	Iijima et al., 2018
Pme_P2_AB_f2	female	Aa	P2	5,801,686,036	SAMD00469791	this study
Pme_P2_AB_f3	female	Aa	P2	5,794,900,048	SAMD00469792	
200213-2	female	Aa	P2	5,076,511,288	SAMD00469786	
200217-3	female	Aa	P2	6,205,205,074	SAMD00469787	
200219-2	female	Aa	P2	5,025,096,026	SAMD00469788	
Pme-P2-1	female	aa	P2	4,920,838,170	SAMD00469780	
Pme-P2-2	female	aa	P2	5,525,999,062	SAMD00469781	
200217-1	female	aa	P2	4,186,168,412	SAMD00469789	
210312c	female	aa	P2	4,824,956,042	SAMD00469790	
Pme-P7-1	female	aa	P7	5,624,669,396	SAMD00469782	
Pme-P7-2	female	aa	P7	5,474,884,780	SAMD00469783	
Pme-P7-3	female	aa	P7	5,398,061,554	SAMD00469784	
Pme-P8-1	female	aa	P8	6,228,670,000	SAMD00469785	

Supplementary Table S2. Search for *doublesex* isoforms by RNA sequencing

Contig	isoform	Sample	Method	Mapping	Length	
STRG.23.1	F1	mesu6	HISAT2/Stringtie	a allele	1777	
STRG.24.1		mesu9	HISAT2/Stringtie	a allele	1777	
STRG29.1		mesu10	HISAT2/Stringtie	a allele	1777	
DN7106_c0_g1_i9	F2	lr860	Trinity		2032	
DN7106_c0_g1_i11		lr860	Trinity		1997	
DN5166_c0_g1_i1	F2’	mesu10	Trinity		986	
DN5166_c0_g1_i2		mesu10	Trinity		986	
CUFF.16318.1		lr860	STAR/Cufflinks	A allele	1532	
CUFF.13950.2	F2’’	mesu10	STAR/Cufflinks	A allele	8130	Exon3: F2; exon4–6: F1
DN3146_c0_g1_i1	F3	mesu6	Trinity		1110	
DN6049_c0_g2_i1		mesu9	Trinity		2323	
DN6049_c0_g2_i4		mesu9	Trinity		2272	
STRG.8.1		mesu6	HISAT2/Stringtie	A allele	1716	
STRG.9.1		mesu9	HISAT2/Stringtie	A allele	1716	
STRG.12.1		mesu10	HISAT2/Stringtie	A allele	1716	
CUFF.16418.1		mesu6	STAR/Cufflinks	a allele	1102	
CUFF.16936.2		mesu9	STAR/Cufflinks	a allele	5485	
CUFF.15700.1		mesu10	STAR/Cufflinks	a allele	5435	
CUFF.14600.2		mesu6	STAR/Cufflinks	A allele	8630	
CUFF.14850.1		mesu9	STAR/Cufflinks	A allele	8487	
CUFF.13950.1		mesu10	STAR/Cufflinks	A allele	7975	
CUFF.13868.1	male	osu20	STAR/Cufflinks	A allele	718	
CUFF.14350.1		osu22	STAR/Cufflinks	A allele	4390	

Supplementary Table S3. Lists of qPCR primer and siRNA

a qPCR primer

Target gene	qPCR primer name	Formard (5'–3')	Reverse (5'–3')
dsx-A (P. memnon)	Pme_dsx_mimetic_exon6_1	TGAACACACATTATACGCGAG	GCTACACAACACCGTCAAAG
dsx-a (P. memnon)	Pme_dsx_a_spec_qPCR	AAAGTGGCGGTTGAACCAAA	TTCAATTGTTCGCTGAGACCAT
dsx-H (P. polytes)	Pp_dsx_H_spec_qPCR	GCTGCAACTCACCACGCAGCGTCACA	CCGCGCTCGGAGTCGACGGAGGT
dsx-h (P. polytes)	Pp_dsx_h_spec_qPCR	GCTGCAACTTACCACGCGGCGCAACT	CCGAGCTCGAAGTCGACGGGGGC
RpL3	Pp_rpl3_qPCR-F2	CACAAAGGGCAAGGGATAC	ACAAGCTACTTTACGCAGAC

b siRNA

siRNA target gene	siRNA name	siRNA Target sequence	Sense (5'–3')	Antisense (5'–3')
dsx-A & *dsx-a*	Pp_dsx_common	TTGGTGGAGAACTGTCACAGACT	GGUGGAGAACUGUCACAGACU	UCUGUGACAGUUCUCCACCAA
dsx-F (female isoform specific)	Pme_dsx_F	AAGCACAATTTGAACATCTTTGA	GCACAAUUUGAACAUCUUUGA	AAAGAUGUUCAAAUUGUGCUU
dsx-a (non-mimetic specific)	Pme_nonmimetic_ORF	TGCGCCCGTTGTCGTAATCATCG	CGCCCGUUGUCGUAAUCAUCG	AUGAUUACGACAACGGGCGCA
